# Scaffolds for Pelvic Floor Prolapse: Logical Pathways

**DOI:** 10.1155/2018/8040893

**Published:** 2018-02-01

**Authors:** Julio Bissoli, Homero Bruschini

**Affiliations:** Hospital das Clínicas da Faculdade de Medicina da Universidade de São Paulo (HCFMUSP), 05410-020 São Paulo, SP, Brazil

## Abstract

Pelvic organ prolapse (POP) has borrowed principles of treatment from hernia repair and in the last two decades we saw reinforcement materials to treat POP with good outcomes in terms of anatomy but with alarming complication rates. Polypropylene meshes to specifically treat POP have been withdrawn from market by manufactures and a blank space was left to be filled with new materials. Macroporous monofilament meshes are ideal candidates and electrospinning emerged as a reliable method capable of delivering production reproducibility and customization. In this review, we point out some pathways that seem logical to be followed but have been only researched in last couple of years.

## 1. Introduction

Greeks created the term “prosthesis” or “to place before.” Hernia repair usually consists of placing meshes to assist the suture control of protrusions [1]. In modern history, Theodore Billroth in 1857 inspired all prosthesis designers with his proposition “If we could artificially produce tissue of the density and toughness of fascia and tendon, the secret of the radical cure of the hernia repair would be discovered” [2], but its concept of repair dates back to Greeks who first described silver strands woven sutured with gold wire to act as a prosthesis [3].

Plastic development revolutionized hernia repair firstly with nylon woven prosthesis, abandoned due to loss of strength caused by hydrolysis, followed by other materials as polypropylene (PP), polytetrafluorethylene (PTFE), Dacron, and polyethylene (PE) [4].

In anatomical terms, hernias are quite similar to pelvic organ prolapse where one or more vaginal compartments descend downwards vaginal opening causing vaginal bulging [5] and this similarity brought the same treatment concepts from one to another [6–8]. What was ignored was the thin layer of mucosae capable of covering an occasional prosthesis, the elasticity inherent to the organ (specially during sexual activity), and exuberant vaginal local florae [5, 7].

## 2. Pelvic Organ Prolapse

In recent years, development of a new kind of mesh specifically designed for vaginal surgery earned some interest because of a major withdraw from mesh market by leading industries after FDA safety warnings on complications of polypropylene in prolapse surgery and due to massive losses on court due to litigation [8–10].

POP is a high prevalent disease occurring in up to 37% of asymptomatic women [11], with lifetime risk of intervention up to 80 years estimated to be 11 to 20% [12, 13] and reoperation rates due to symptoms around 30% [14].

With such high prevalence and failure rates with conventional treatments up to 56% [15], it was a natural movement for doctors and researchers to start developing reinforcements to sutures performed during vaginal compartments treatment.

## 3. Reinforcements Subtypes

The materials used to reinforce POP treatment can be autologous, heterologous, synthetic absorbable, or inabsorbable [7]. The initial tests with fascia lata, acellular dermis, and rectus sheet did not show superior results to conventional techniques, with 38% failure [16], and are limited by donor site, pain, surgery time increase, and quality/quantity variable [17, 18] with the clear advantage to not trigger any immune response. Heterologous grafts keep the same or inferior results potentially acting as a carrier to viral and prion diseases [7, 8].

Among synthetic reinforcement, the polyglactin is shown to be absorbed without matrix remodelling and failure before 2 years of implant [19–21] while inabsorbable polypropylene (most widely used) showed 80% success compared to traditional techniques in short time [22] but with midterm complications such erosions/extrusions around 25% [23] causing FDA to release safety warnings in 2008 and 2011 [9, 10] triggering ethical legal problems and prompting leading companies to withdraw from market [8].

Considering limited results with classical approaches and materials, especially after withdrawing of synthetic meshes manufacturers, a blank space emerged to be filled with new materials.

## 4. Synthetic Meshes Classification

Meshes are a subtype of synthetic matrices with organized woven or knitted pattern. Amid in 1997 classified meshes accordingly to its porosity and filaments structure [25], predicting complications as bigger pores allow higher vascularization, fibroblasts ingrowth, and immune cells infiltration increasing biocompatibility and infection resilience [26]. On the other way, multifilament fibres (with space between filaments inferior to 10 microns) and microporous meshes are less susceptible to cellular ingrowth and macrophage/lymphocyte action (they measure around 9–20 microns) being prone to bacterial colonization.

Theoretically, an ideal material would be a macroporous monofilament type I mesh of Amid [27–29] and practical applications confirm such statement with types II, III, and IV being used for POP and SUI with short-term complication rates around 20–30% [22, 27, 30].

## 5. Synthetic Matrices Production

There are several methods for higher porosity matrices production, like self-assembly [31], phase separation, solvent casting and particulate leaching, freeze drying, melt moulding, gas foaming, and solid free-forming [32]. All of them with reproducibility limitations and poor control of characteristics such diameter of fibres and pore size, pore geometry, and fibre orientation with electrospinning as an alternative to surpass all these difficulties [33].

## 6. Electrospinning

Electrospinning is a physical phenomenon observed when a high viscosity polymer solution is exposed to an intense electric field in a recipient where the liquid is extruded in a slow fashion through one small or multiple small orifices (i.e., spinneret). Usually such solution is made by a high molecular weight polymer in a solvent with high vapour pressure (i.e., capable of easily evaporate in a room temperature) and low conductivity with high dielectric constant (i.e., high resistance to stress before brake and allow passage of electric current) [34].

Besides relatively high viscosity of material used, under high electric field produced by a high tension power source, a superficial tension rupture at the tip of the spinneret occurs (usually a blunt tip needle). This rupture creates an instability region causing stretching of viscous solution towards an earthed collector, parallel to the lines of force of electric field, producing micro/nanometric scale fibres in diameter. Such fibres become dried after evaporation of solvent during its pathway to the collector.

This process is known since the beginning of the last century being observed by Rayleigh in 1897, detailed by Zeleny in 1914, and patented by Formhals in 1934 for textile production. Taylor, in his studies about electrostatics (1969), described the jets produced by the technique (today called by his name, Taylor's cone), but electrospinning as a biomedical application emerged only after the 1990s because of its price and reproducibility, being one of the commonest methods of matrix production in bioengineering.

Parameters controlled during electrospinning process are classically divided into solution properties, controlled variables, and environmental parameters [32]. The solution properties included viscosity, superficial tension, conductivity, and molecular weight, while controlled variables are flow rate, electric field intensity, collector distance to the end of the tip of spinneret, size of the tip, and geometry of collector. Environmental parameters are temperature, pressure, and air speed [34].

From all described variables, the most important is the solution concentration to determine, among other things, the diameter of fibres.

## 7. Polymers

Polymers are big molecules produced by repetition of numerous subunits called monomers and depending on the number of types of repetitive units can be further classified as homopolymers or copolymers. Its name derives from Greek* polus* (i.e., lots of) and* merossu *(i.e., parts), and its physical characteristics are dependent on the size and length of their chain. Usually as polymer chain increases, its degradation time is prolonged, and its viscosity, strength, and rigidity are higher, being common to allude to it in terms of molecular weight (minimum, maximum, and average value) [35].

**Figure 1:**
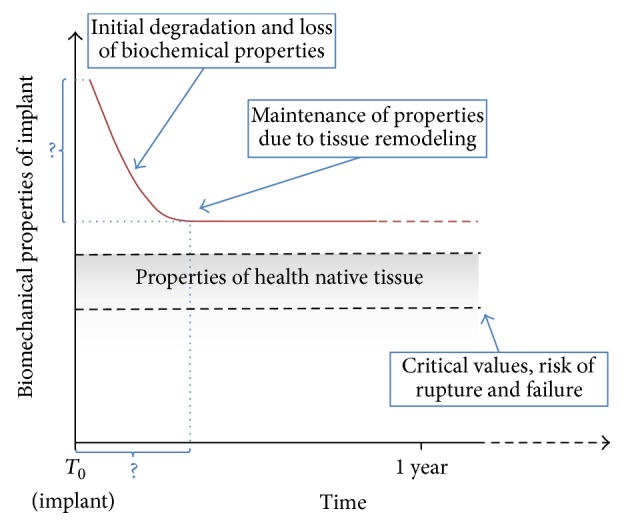
Ideal biomechanical properties of biodegradable scaffolds (adapted from Osman et al. [24]).

They can be further subclassified as naturals or synthetics and absorbable or inabsorbable. For bioengineering applications, there are necessary characteristics: their nontoxicity (direct or indirect), nonimmunogenicity, noncarcinogenicity, and biocompatibility (ability to integrate with living tissues). Desirable characteristics are resilience to infection, low cost, ability to be easily manufactured and stored, and absorption/degradation in a fashion that allows repopulation and integration by living cells from target tissue and mechanical properties compatible with target tissue function from day 0, during repopulation until its complete reabsorption (Figure 1) [24].

For each target tissue the desirable properties are different. For each polymer used in an implant, a drop in its mechanical properties will occur proportionally to its reabsorption and thus depends on its degradation speed and its micro/nanostructure. Ideally, such characteristics to be mimic should be known previously (ultimate tensile strength, elasticity, and absorption time) and adjusted accordingly to each new microenvironment.

Among the most common polymers electrospun are polylactic acid (PLA), polyglycolic acid (PGA), polyhydroxybutyrate covalerate (PHBV), polycaprolactone (PCL), chitosan, collagen, and polyurethanes (PU).

Aliphatic polyesters like polycaprolactone and polyglycolic acid are materials extensively known since 1960 because of its biocompatibility. They are hydrolysed and/or enzymatically digested to nontoxic subproducts. They usually have high elastic modulus and tensile strength with poor elongation (considered rigid polymers), great candidates for tissue engineering [30].

Polyhydroxyalkanoates as poly(3-hydroxybutyrate-co-3-hydroxyvalerate) (PHBV) are recently investigated in tissue engineering because of its resilience to infection and hydrolysis much like polyurethanes (PU) that additionally have calcification resistance and are known in applications as synthetic rubber for more than 30 years. Both present longer absorption times when compared to polyesters being PU known by its great ability to cope with strain.

## 8. Biomechanical Tests

There are no standard protocols for biomechanical testing of vaginal tissues, being the best methodologies derived from uniaxial tests (i.e., test performed only in one direction), measured in a tensiometer stress-strain curves. Multiaxial tests or biaxial tests potentially would reflect a model closer to its real mechanical properties, but they are more complex and demand specific software and special equipment making its standardization even more complex than uniaxial tests.

Briefly, the uniaxial biomechanical test consists of firmly securing sample between two clamps connected to a tensiometer and a computer and distending the sample in a controlled fashion. Previous known distance of clamps, sample width and thickness, and constant uniaxial distention of clamps (i.e., keeping the same orientation and speed of force) until sample failure or test manually stops will provide data to calculate its biomechanical characteristics.

The ultimate tensile strength is calculated dividing the load applied to the sample (in Newtons, N) by the cross section area of the sample being reported in N/m2 (Pascals). Strain is calculated dividing elongation of the sample (in meters, m) by its initial length between clamps (in meters, m) resulting in a quotient without units or a percentage. Commonly, such data is plotted on a graph and shows a linear portion where tension is direct proportional to strain (respecting Hooke's Law) where strain is reversible or elastic and then a plateau where elongation is irreversible or plastic followed by an inflection (ultimate tensile strength) with correspondence to *X*-axis defining maximum strain.

**Figure 2:**
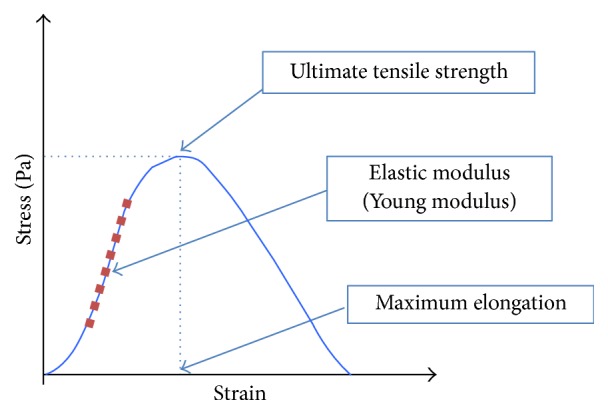
Stress-strain example curve with elastic modulus, ultimate tensile strength, and maximum elongation.

The example can be observed at Figure 2, the elastic modulus or Young modulus can be calculated from inclination of linear portion of the stress-strain curve and it is inversely proportional to elasticity of the sample.

To develop an ideal material for a target tissue, knowledge of its properties is crucial and a few studies showed biomechanical properties for human tissues. The vast majority do not normalize its data using cross section area exhibiting values only in Newtons, preventing posterior comparisons [7, 36, 37].

## 9. Paravaginal Mechanical Properties

Choe et al. compared 2 × 5 cm strips of fascia lata, dermis, rectum sheet, and vaginal mucosae (measure commonly used at sling surgeries [38]) in women operated for various reasons [39] showing that fascia lata had the biggest tensile strength (217 N), followed by human dermis (122 N) and rectum sheet and vaginal mucosae (both with 42 N).

Lei et al. analysed 43 women after hysterectomy and categorized them in groups, premenopause and postmenopause and with or without prolapse (Table 1), testing with uniaxial tests tissues with 5 mm × 25 mm and plotting their stress-strain curves establishing native values considered maximum (premenopause) and minimum (postmenopause) obtaining maximum elongation and elastic modulus references for most publications in this field by being comparable to other samples (i.e., normalized by cross section area) [40]. As a critic to such conclusions it is important to notice that samples tested were from vaginal mucosae closer to apex and not from suspensory ligaments or mucosae closer to cystocele/rectocele common defects.

## 10. Discussion

We do not know exactly how much is the demand in Newtons for the pelvic floor, but we estimate forces acting on it to be around 2,2 to 13,4 N/cm from stand still to stand with Valsalva [41]. Meanwhile, we know that the best autologous candidate—rectum fascia—could cope up to 16 N/cm with a 25% vertical strain [42].

It is important to highlight that tensile strength alone is not capable of predicting success in reconstructive urogenital surgery, and fascia lata and acellular dermis (both quite strong) also have high relapse rate before 2 years of surgery [19, 43] showing that, for biocompatible absorbable materials, remodelling in the host is probably of higher importance than the initial tensile strength of the implant [44].

We still do not have a substitute for paravaginal weakened tissues with native characteristics in terms of resistance and flexibility and data have shown that we are not close to find it [9, 10, 23, 45], notably a potential replacement for polypropylene meshes used in POP and SUI surgery, efficient by anatomical point of view but with complications in POP case that precludes its use.

Such complications have multiple factors involved, but polypropylene high resistance, inflexibility, and inelasticity certainly play a role heightened by a contraction tendency after the implant, despite being considered biocompatible [8].

Several good candidates had been prospected to replace it, each scaffold with or without specific cell type being regarded as ideal. Oral fibroblasts [46], adipose derived stem cells [47], vaginal fibroblasts [48], and muscle cells [49] all have its qualities and defects being more similar or not to targeted paravaginal tissue, being more easily or not to harvest and/or to cultivate onto scaffolds produced with variations of PLA, PLGA, PU, and processed small intestine submucosae.

Considering costs, regulations, and facilities needed to widespread cell culture to clinical practice, probably the first material produced to replace polypropylene meshes in POP practice will be an off-the-shelf synthetic scaffold with great cell affinity targeted to a specific biomechanical demand of paravaginal tissue.

Animal experiments of at least 6–12 months in a model physiologically relevant to POP will help to establish how degradation and neotissue formation will affect properties of such material.

## 11. Conclusion

We have been done a lot to replace polypropylene meshes by matrices not only capable of withstanding tension but also capable of interacting with cells and promote tissue remodelling with fibroblasts ingrowth, extracellular matrix production, and angiogenesis [50] and we already made some progress in this regard with POP.

Respecting Billroth's principle and producing materials strong enough but sufficiently elastic to allow natural distention of vaginal tissue [44] and biocompatible to reflect paravaginal properties [51] are the right pathway and surprisingly only recently started to be followed.

## Figures and Tables

**Table 1:** Elastic modulus, maximum elongation, and ultimate tensile strength in women with and without prolapse (adapted from Lei et al.).

	Control premenopause	Prolapse premenopause	Control postmenopause	Prolapse postmenopause
Elastic modulus (MPa; mean ± EPM)	6.65 ± 1.48	9.45 ± 0.70	10.26 ± 1.10	12.10 ± 1.10
Maximum elongation (mean ± EPM)	1.68 ± 0.11	1.50 ± 0.02	1.37 ± 0.04	1.14 ± 0.06
Ultimate tensile strength (MPa; mean ± EPM)	0.79 ± 0.05	0.60 ± 0.02	0.42 ± 0.03	0.27 ± 0.03

## Additional Points

*Highlights*. (i) Pelvic organ prolapse treatment borrowed anatomical concepts from hernia repair without considering local differences in terms of elasticity, florae, and sexual activity. Major brand meshes for POP have been withdrawn from market due to complications and litigious problems. (ii) Despite the logic to know that the material is to be replaced or reinforced and then use this data to develop a new prosthesis, pelvic prolapse lacks in terms of basic research of biomechanical properties and lacked in safety studies before applying polypropylene meshes for this purpose. (iii) To promote true remodelling and cure we not only should think about reinforcement and biomechanical properties but also must develop a material that interacts with cells and promote fibroblast ingrowth and extracellular matrix production. Lack of these combined qualities invariably will lead to failure.
